# Titta: A toolbox for creating PsychToolbox and Psychopy experiments with Tobii eye trackers

**DOI:** 10.3758/s13428-020-01358-8

**Published:** 2020-03-03

**Authors:** Diederick C. Niehorster, Richard Andersson, Marcus Nyström

**Affiliations:** 1 Lund University Humanities Lab and Department of Psychology, Lund University, Box 201, SE-221 00, Lund, Sweden; 2 Tobii Pro AB, Tobii Pro AB, Box 743, 182 17, Danderyd, Sweden; 3 Lund University Humanities Lab, Lund University, Box 201, SE-221 00, Lund, Sweden

**Keywords:** Eye tracking, Eye movements, Stimulus creation, Equipment interface

## Abstract

We present Titta, an open-source toolbox for controlling eye trackers manufactured by Tobii AB from MATLAB and Python. The toolbox provides a wrapper around the Tobii Pro SDK, providing a convenient graphical participant setup, calibration and validation interface implemented using the PsychToolbox and PsychoPy toolboxes. The toolbox furthermore enables MATLAB and Python experiments to communicate with Tobii Pro Lab through the TalkToProLab tool. This enables experiments to be created and run using the freedom of MATLAB and Python, while the recording can be visualized and analyzed in Tobii Pro Lab. All screen-mounted Tobii eye trackers that are supported by the Tobii Pro SDK are also supported by Titta. At the time of writing, these are the Spectrum, Nano, TX300, T60XL, X3-120, X2-60, X2-30, X60, X120, T60 and T120 from Tobii Pro, and the 4C from Tobii Tech.

**Electronic supplementary material:**

The online version of this article (10.3758/s13428-020-01358-8) contains supplementary material, which is available to authorized users.

## Introduction

Eye trackers are used to record where people look and how their eyes move in one of multiple reference frames (see Hessels et al., 2018). These devices are used by an ever-increasing number of researchers in a wide array of academic fields as well as industry (Holmqvist et al., 2011). To record eye movements using eye trackers, either complete graphical software packages provided by the eye tracker’s manufacturer or third parties are used, or tools developed by academics that interface the eye-tracker software development kit (SDK) with high-level programming languages of their choice (e.g. Cornelissen 12 et al., 2002; and Niehorster and Nyström 2019). The manufacturer software provides an easy to use graphical interface that is however often also limited in functionality, such as offering support for only picture and video stimuli and limited trial randomization. As such, the latter programming tools are often the only option for advanced researchers with specific needs not supported by the manufacturer’s software packages. However, such a flexible system-specific package is not available for eye trackers from Tobii, one of the large manufacturers of eye trackers for researchers. While Tobii eye trackers are supported by tools that offer a generic programming interface to a wide variety of eye trackers, such as PyGaze (Dalmaijer et al., 2014) or the ioHub library that is part of PsychoPy, the drawback of such generic interfaces is that they only provided limited access to advanced system-specific capabilities of some eye trackers. Examples of these system-specific capabilities are provided in the “Implementation” section.

In this article, we therefore present Titta,^1^ a Tobii- specific software package that allows for easy integration of Tobii eye trackers with experiments written in MATLAB with PsychToolbox (Pelli, 1997; Brainard, 1997; Kleiner et al., 2007) and in Python with PsychoPy (Peirce^2007^, ^2009^), while providing full access to all features of each of the supported eye trackers. Titta is built upon the C and Python versions of the low-level Tobii Pro SDK and, amongst other features, provides an easy to use participant setup, calibration and validation interface that is implemented directly in PsychToolbox or PsychoPy drawing commands. Titta can be integrated into existing experiments by adding only a handful of lines of code, but at the same time also enables access to all setup and operational features of the supported Tobii eye trackers. The PsychoPy version of Titta furthermore supports PsychoPy builder (Peirce et al., 2019), allowing easy integration of Tobii eye trackers in experiments built with this graphical experiment builder. Titta is available from https://github.com/dcnieho/Titta (MATLAB) and https://github.com/marcus-nystrom/Titta(Python).

Once eye movements are recorded, researchers may want to look through their recordings by means of replays or other visualizations, and perform analysis of their eye movement data. Such functionality is not provided by Titta, which only provides an interface for operating the eye tracker. The Tobii Pro Lab software package provides such replay and analysis functionality. At the time of writing, Tobii Pro Lab however provides only basic stimulus creation options, making it not suitable for many experimental paradigms. Such software packages are furthermore unlikely to ever cater to the custom needs of all researchers. For instance, niche needs such as the non-aging foreperiods recommended in antisaccade protocols (Antoniades et al., 2013) are unlikely to be provided. As such, researchers often have to choose between tools with a graphical user interface that also provide data vizualization and analysis functionality but support a limited set of research paradigms, and programming environments that allow full flexibility but require the user to write their own tools for data visualization and analysis. To remedy this situation, Tobii Pro Lab provides an “External presenter’’ project type that enables external programs to control Tobii Pro Lab through a remote control interface. Using this mode, researchers can use any means of creating and running their experiment, while at the same time instructing Pro Lab to record eye movements and informing it of what stimuli were shown on the screen. This provides researchers with full flexibility in developing experimental paradigms, while also enabling them to use Pro Lab’s visualization and analysis capabilities. The Titta toolbox provides the TalkToProLab tool which implements a convenient wrapper for Tobii Pro Lab’s External Presenter interface for MATLAB and Python. TalkToProLab, which operates independently of the Titta tool, is also presented in this article.

## Tobii eye trackers

In its almost 20-year long existence, Tobii has produced a range of eye trackers. Most of these eye trackers have been so-called remote eye trackers, i.e., systems that are usually used attached to a computer screen and allow some freedom of movement of the observer in front of the screen (but see Hessels et al., 2015; and Niehorster et al., 2018). Titta supports all the remote eye trackers that are supported by the Tobii Pro SDK. At the time of writing, these are the Spectrum, Nano, TX300, T60XL, X3-120, X2-60, X2-30, X60, X120, T60 and T120 from Tobii Pro, and the 4C from Tobii Tech. Titta does not support the Tobii eye trackers integrated in VR headsets. Furthermore, Tobii’s head-worn eye trackers such as the Tobii Pro Glasses 2 are not supported. For the latter device, users are referred to other tools (e.g. De Tommaso and Wykowska 2019; and Niehorster et al., 2020). 

The supported Tobii eye trackers span a wide range of sampling rates and data quality. The supported eye trackers range from low sampling rate entry models such as the Tobii Pro Nano (60 Hz) and X2-60 (60 Hz), to systems such as the TX300 and Spectrum that compete with the state-of-the-art eye trackers from other manufacturers in terms of data quality (Nyström et al., 2018) and that provide gaze position data sampled at up to 1200 Hz.

## Implementation

Two parallel versions of Titta and TalkToProLab have been developed. One is implemented in MATLAB and uses a MATLAB extension (MEX) file for parts of its implementation, and relies on PsychToolbox (Pelli, 1997; Brainard, 1997; Kleiner et al., 2007) for its graphical setup interface. The other is implemented as a native Python class that is compatible with Python 3.6 and uses PsychoPy to draw its graphical setup interface. Titta has been tested with Tobii Pro SDK version 1.7.0.2. Both the MATLAB and the Python versions of Titta support 32-bit and 64-bit environments. Main testing and development was done on Windows, but use on Linux is also supported.

Full documentation and a complete listing for the programming interface (API) for Titta is provided in the readme.md file in the Titta distributions at https://github.com/dcnieho/Titta (MATLAB) and https://github.com/marcus-nystrom/Titta (Python). The toolboxes are furthermore highly configurable, a full overview of their settings is also provided in these readme.md files. In the below sections, we describe how key functionality of Titta was implemented and is expected to be used. Function calls in the text are provided in the camelCase naming convention used in the MATLAB version of the toolbox. The Python version has the same functions with the same names, though they are spelled using snake_case (e.g., Titta.sendMessage() and Titta. send_message()). Calls to methods or properties of the Titta class are denoted as Titta.methodOrProperty, and likewise for the TalkToProLab class: TalkToPro Lab.methodOrProperty. Note that all methods of the Titta and TalkToProLab classes except Titta. calibrate() are implemented in pure MATLAB and Python, and as such can be used without PsychToolbox or PsychoPy.

### Participant setup

**Fig. 1:**
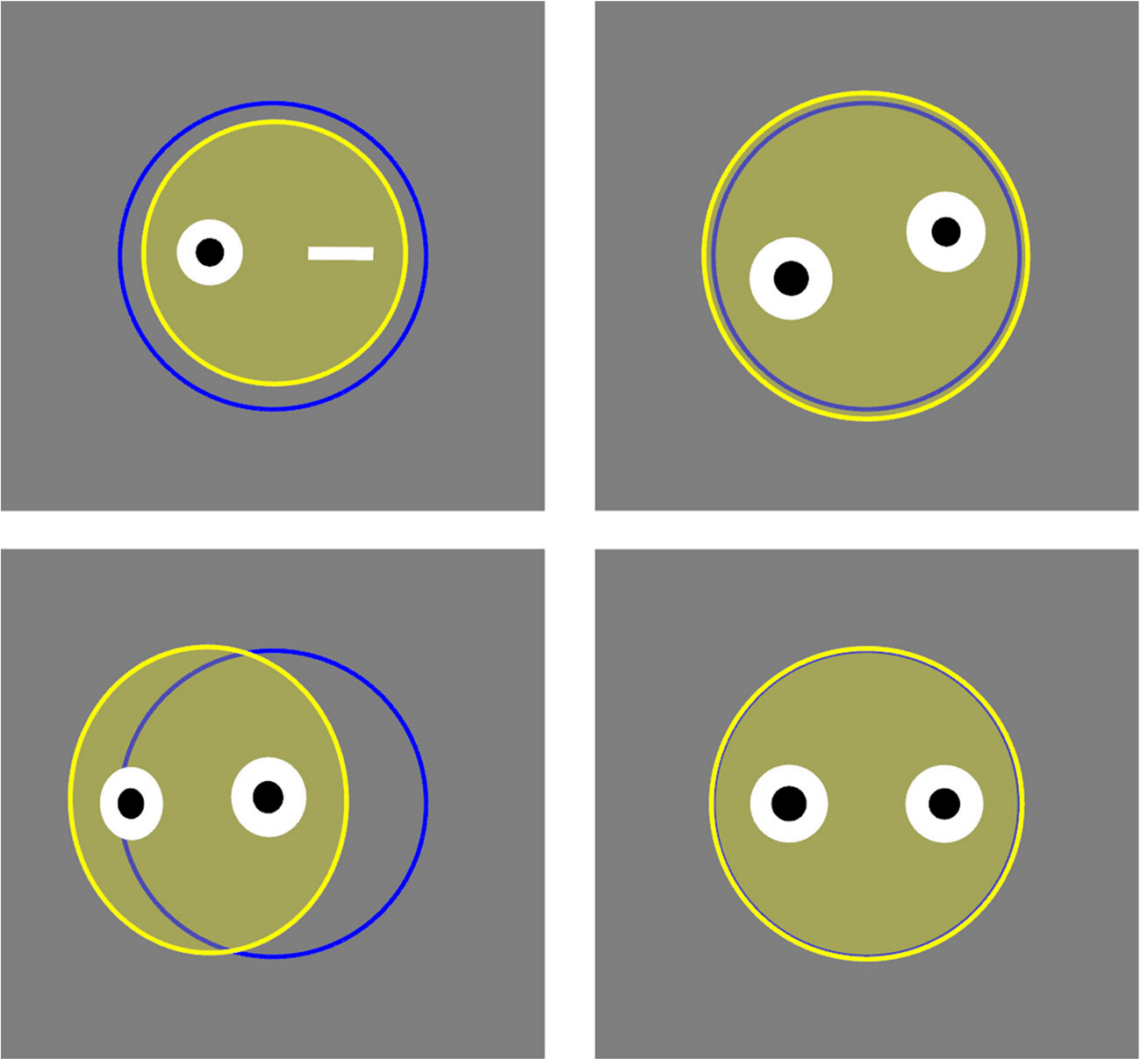
**The head display in the participant setup interface**. The blue circle indicates the reference position, and the yellow filled circle the participant’s head, along with two eyes and their pupils. The four panels show different situations. Left-top: Participant is too far back and the right eye is not tracked. Right-top: participant’s head is rolled with respect to the eye tracker. Left-bottom: participant’s head is rotated side-ways such that one eye is further away from the eye tracker than the other. Right-bottom: participant is correctly positioned and oriented

When Titta.calibrate() is called, by default first a display is shown that is designed to properly position the participant. This display consists of a blue circle representing a reference position, and a stylized head indicating the current position and orientation of the participant (see Fig. 1). The stylized head display functions like a mirror in that it, for instance, gets smaller when the participant is further back, and a closed eye is drawn when track of an eye is lost. It furthermore by default provides real-time pupil-size data to allow ascertaining whether pupil size of the participant is not extreme and stably tracked. The head display is implemented by the ETHead class, which can be used separately from Titta.

**Fig. 2:**
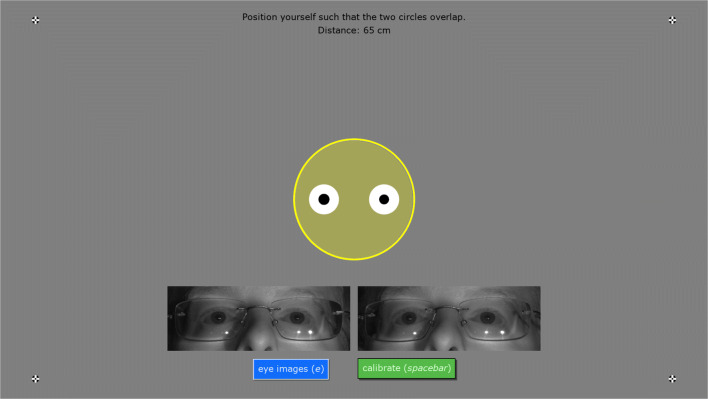
**The participant setup interface**. The blue circle indicates the reference position, and the stylized head represents to position of the participant. Furthermore drawn are a setup instruction to the participant, four fixation targets in the screen corners and eye images (acquired with a Tobii Pro Spectrum)

Using this display, participant setup consists of asking the participant to move such that the head exactly coincides with the blue circle. This is a simple and intuitive task for participants that in our experience often requires little assistance from the experimenter. For experiments requiring precise positioning of the participant, the reference position can be set to a specific position in the eye tracker’s user coordinate system.^2^ If no reference position is provided by the user, depending on the eye tracker it can either be automatically set to the center of the eye tracker’s headbox, or will be drawn based on the eye tracker’s positioning guide stream. If the eye tracker supports providing eye images, these can be viewed on this screen as well by pressing the eye image toggle button (see Fig. 2). Once the participant is correctly positioned, we recommend asking them to look at the four fixation targets in the corners of the screen, to ensure that track is stable across the entire span of the screen.

### Calibration and validation

Once the participant is correctly set up, a calibration can be started. We describe the calibration and validation process and the accompanying interface below, and have made a demo video of the process available online in the Supplemental Material. By default, the calibration screen consists of a fixation target from Thaler et al., (2013, ABC in the lower panel of their Fig. 1), chosen because it was found to minimize drift and yield the lowest microsaccade rate during fixation. This fixation target jumps across the screen to go through a series of calibration points (by default five points laid out in an X-pattern) either in the specified order or randomized (default). Directly after calibration, a further series of validation points is shown to the participant. By default, four validation points laid out in a diamond-shaped pattern are shown in randomized order. The diamond pattern is used to maximize the distance between the validation and calibration points, thereby allowing to judge the worst-case data quality provided by the evaluated calibration.

The look of the calibration and validation screens can be customized by the programmer by providing their own calibration screen drawing function. This allows replacing the default statically displayed fixation points with anything most suitable for the participant group, such as videos of swirling patterns accompanied by sounds to help attract attention. The provided calibration screen drawing function is called for every frame and provided with a command such as ‘draw' or ‘new' to indicate state of the calibration, the ID of the current point to be displayed, its location, a monotonously increasing tick value counting the number of times the drawing function has been invoked and a string indicating whether the participant is currently undergoing calibration or validation. An example implementation of such a drawing function is provided in the AnimatedCalibrationDisplay class that is provided with the Titta toolbox, and is used in the all the demo scripts provided with Titta.

Once the calibration screen is entered, by default the spacebar has to be pressed to start the calibration sequence, after which it runs to completion unattended. Two other modes are also available (controlled by the cal.autoPace setting), one in which the full calibration procedure completes unattendedly, and one in which each calibration point needs manual confirmation by means of a spacebar press. Letting the participant manually start data collection for each calibration point has been found to improve calibration accuracy (Nyström et al., 2013), but this mode can also be used when calibrating participants who cannot be instructed to fixate, such as babies and monkeys. During calibration, the “backspace“ key can be pressed to restart the current calibration point, the “r” key to restart the calibration, and the “escape” key to return to the setup screen. 

**Fig. 3:**
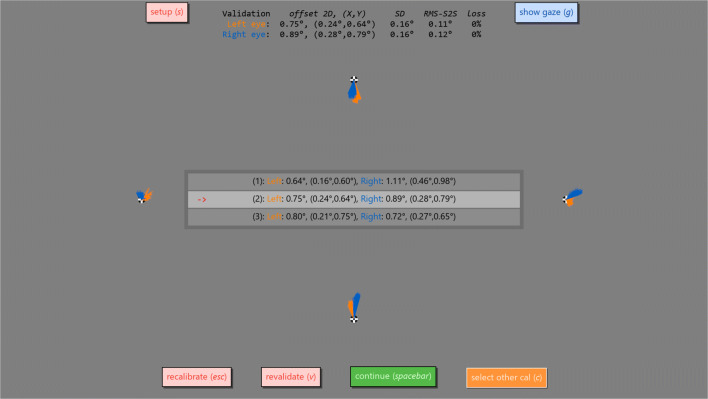
**The validation result screen**. Shown is a graphical representation of the data collected for each of the four validation points. Each sample is drawn as a line from the respective validation point to the recorded gaze position, and is displayed at full scale to make achieved data quality directly intuitable. The *recalibrate* button starts a new calibration and validation sequence, the *revalidate* button performs a new validation for the currently selected calibration and the *setup* button brings the setup screen back up. The *show gaze* button toggles a real-time display of the gaze position reported by the eye tracker (not shown), and the *select other cal* button brings up a menu from which another calibration can be made active if multiple calibrations have been performed for the current session. This menu is shown in the center of the screen. Finally, the *continue* button selects the current calibration as the one to use for the recording session, and closes the participant setup screen, causing Titta.calibrate() to return

Once calibration and validation have been completed successfully, a validation result screen is shown (Fig. 3). This display shows a graphical representation of the gaze position data collected during validation (by default 500 ms for each validation point). This graphical representation is drawn at full scale so that the experimenter has a direct representation of what accuracy (deviation in recorded gaze position) was achieved for each validation point in terms of distance on the screen. To further visually judge data quality, experimenters can switch on a visualization of the real-time gaze position reported by the eye tracker.

The validation result display furthermore provides numerical representations of the data quality achieved overall, and for specific validation points when hovering over them with the mouse. For data quality measures (see, e.g., McConkie 1981; and Niehorster et al., 2020 for operationalizations and discussions of data quality measures) for operationalizations and discussions of data quality measures), mean deviation of recorded gaze from the calibration point is shown to denote the achieved accuracy of the calibration. Furthermore, to gauge the precision of the recording, the root mean square of sample-to-sample distances in reported gaze position (RMS-S2S) and the standard deviation of the collected gaze positions (STD) is calculated. Lastly, the amount of data loss is calculated as the percentage of recorded samples for which no valid gaze position was available. These figures are displayed prominently in the interface because we think it is important that researchers are aware of the various aspects of data quality and can directly judge whether data quality is sufficient for their experiment, so that subsequent analysis would yield valid results. The displayed data quality values are also stored in a session’s messages (see “Synchronization”) for permanent storage. It is highly recommended that these messages are saved alongside the eye tracking data (e.g., using Titta.saveData()), so that information about data quality can be retrieved during analysis and reported in scientific publications derived from the recording. Information about all calibrations that were performed during a session can be retrieved from the Titta.calibrateHistory property, and gaze data is recorded throughout the calibration-and-validation process so that the interested researcher can examine these themselves.

From the validation result screen, it is possible to redo the validation of a calibration (e.g., if it is suspected that the participant did not accurately fixate one of the fixation points and the validation results are thus not representative of calibration quality) or to do another calibration and validation sequence. As we have noted previously (Niehorster & Nyström, 2019), we recommend calibrating a participant multiple times and then selecting the best calibration given that in our experience, especially for inexperienced participants, the first calibration often is not the best that a participant can achieve. The validation result screen allows selecting which of the multiple calibrations to use for the recording.

Some Tobii Pro eye trackers (at the time of writing only the Spectrum) support performing monocular calibrations. Titta implements support for doing calibrations of only a single eye, as well as bi-monocular calibrations where the two eyes are calibrated separately in turn as may be useful for research into binocular coordination (see, e.g., Nuthmann and Kliegl 2009; Liversedge et al. 2006; and Švede et al. 2015). Such bi-monocular calibration functionality is implemented as an option in the readme demo experiment.

### Real-time data streams

Tobii eye trackers provide multiple data streams that can be listened to during an experiment. All Tobii eye trackers provide a gaze data stream providing information about the gaze direction of the participant, their pupil diameter, and the location of their eyes in the space in front of the eye tracker. All Tobii eye trackers furthermore provide a stream with information about the process that synchronizes the eye tracker’s clock to the experiment computer’s clock. Depending on supported capabilities, Tobii eye trackers may furthermore provide an eye image stream and an external signal stream that logs activity on the eye tracker’s TTL ports. Each of these streams can be recorded using Titta, with Titta.buffer.start(‘streamname') when using MATLAB and Titta.start_recording (gaze_data=True) when using Python. Note that recorded data is not automatically saved to file, as the reader may be used to from SMI and SR Research eye trackers. See “File saving” for information about how to save data.

When instructing Titta to record data from the gaze stream, or any of the other streams, the data from this stream also automatically becomes available to the experiment in real-time. While for the Python implementation of Titta this data is directly available as a list that the user can manipulate themselves, this was not possible in MATLAB due to restrictions in the MATLAB language. As such, in MATLAB the Titta.buffer interface has to be used. Full documentation of this interface is provided in the readme.md file in the Titta repository under the TobiiMex header, here we will discuss key functionality. In MATLAB, data from any of the streams can be accessed in two ways: consuming and peeking. A consuming access returns the requested data and removes it from the buffer. A peek access on the other hand returns the same data but does not remove it from the buffer. If real-time data access is required but all data should also be stored for later offline analysis, it is recommended that the peek functionality is used so that the buffer contains all data recorded during the session. Both the consume and the peek functionality is accessible through two different interfaces, i.e., either a specific number of samples can be requested using Titta.buffer.consumeN() and Titta.buffer. peekN(), or all the data between two timestamps using Titta.buffer.consumeTimeRange() and Titta.buffer.peekTimeRange().

### Synchronization

To allow analysis of the recorded gaze position signals, it is of critical importance that the times at which events happen during the experiment program (such as stimuli being shown on the screen or key presses being registered) can be related to specific episodes in the recorded eye-tracking data. This is typically done by keeping a timestamped log file consisting of messages denoting when an event of interest occurred on the experiment computer. It is then still required to acquire data from the eye tracker that is timestamped in the same time base as the experiment computer’s log file. The Tobii Pro SDK takes care of providing data from the eye tracker with timestamps that are synchronized to one of the experiment computer’s clocks.^3^ Titta provides the message log functionality through the Titta.sendMessage() function. This function will create its own timestamp for the message reflecting the time the Titta.sendMessage() function was called. When used with PsychToolbox, Titta.sendMessage() can also be provided with the timestamp of an event signaled by PsychToolbox (e.g., a window Flip or keypress). The participant and setup interface invoked by Titta.calibrate() produces a series of log messages that amongst other things indicate when each calibration or validation point is shown, the final calibration chosen by the researcher to be used for the recording session, and the data quality calculated from each validation. Note that the logged messages are not saved to file automatically, as the reader may be used to from SMI and SR Research eye trackers. See “File saving” for information about how to save data. 

### File saving

In contrast to the SMI and SR Research eye trackers that save gaze data directly to a file, Tobii eye trackers only provide data streams through the Tobii Pro SDK. Tobii trackers also do not handle user-provided log messages. Users of the Tobii Pro SDK, and by extension Titta, are responsible themselves for storing the data collected during a session.

Titta provides the programmer with multiple options. First, Titta.saveData() can be used to save all data from a recording session directly to file. The data stored includes the content of all stream buffers, all the logged messages, information about all the calibrations that were performed, as well as information about the eye tracker and its setup and the Titta settings used for the recording session. Secondly, the function Titta.collectSessionData() can be used to obtain all the above data, that the user can then store to file themselves, perhaps alongside other output from their experiment. Finally, programmers can directly consume individual data streams and save these to file for more flexible applications.

### Separate operator screen

**Fig. 4:**
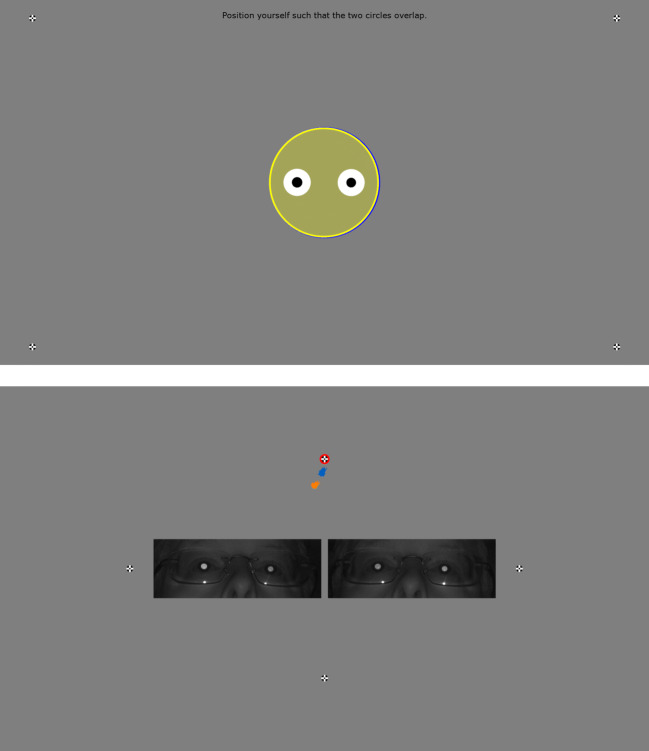
**Setup and calibration interface when using a separate operator screen**. Top panel: simplified setup screen shown to the participant when a separate operator screen is available (cf. Fig. 2). Bottom panel: operator view during calibration and validation. The fixation target that is currently shown to the participant is highlighted with a red circle, gaze data from the last 500 ms is shown for the two eyes (groups of blue and orange dots) and the eye images provided by the eye tracker are also shown if available (the images in this figure were acquired with a Tobii Pro Spectrum)

In some situations, experiments using an eye tracker require a separate operator screen. Examples of this is when working with difficult participant groups, such as monkeys and infants, where a manual calibration procedure controlled by the operator has to be performed, or paradigms where participants should not be aware that they are being eye tracked, such a mental imagery research (Johansson et al., 2006, 2012). Titta supports using a second screen that is connected to the experiment computer as an operator screen. When using a separate screen for the operator, the participant is presented with a minimal interface during setup (Fig. 4, upper panel), and the validation result screen is shown on the operator screen. The participant is shown the message “Please wait...” while the experimenter can examine the calibration quality on the validation result screen. On the validation result screen, the operator has the option to view real-time gaze information. When this functionality is activated, four fixation targets appear in the corners of the participant screen so that data quality can easily be checked across the entire screen, but the real-time gaze data is only shown on the operator screen. To enable performing a manual calibration procedure with participants that cannot be instructed to fixate, the operator screen contains an real-time gaze display as well as eye images if the eye tracker provides them (Fig. 4, lower panel) during calibration and validation.

### TalkToProLab: integration with Tobii Pro Lab

The Titta distribution also provides TalkToProLab, a wrapper around the External Presenter interface of Tobii Pro Lab (tested with Pro Lab version 1.118, may work with version 1.111 and newer). Using this interface, programmers can create their experiment in MATLAB or Python but still use Tobii Pro Lab for visualization and analysis of recordings. This enables programmers to use the full flexibility of these programming environments to create any trial and block randomization as well as any stimulus that they wish, without sacrificing access to the data visualization and analysis capabilities of Pro Lab.

TalkToProLab acts like a remote control for Tobii Pro Lab. Specifically, it currently allows to add a new participant to the External Presenter project that is opened in Pro Lab, to add a recording to a participant, to upload still images or video stimulus material to Pro Lab along with areas of interest (AOIs), and to start and stop a recording. Importantly, TalkToProLab furthermore implements an interface for informing Pro Lab when a stimulus was shown (TalkToProLab.sendStimulusEvent()) and for informing Pro Lab of other events of interest that happened (TalkToProLab.sendCustomEvent()). TalkToProLab fully supports setups where Tobii Pro Lab and the MATLAB or Python experiment software run on the same computer. TalkToProLab’s implementation furthermore is compatible with multi-computer setups where the experiment and Tobii Pro Lab run on different machines, but in this case the programmer themselves has to perform additional synchronization between the experiment and the Tobii Pro Lab machines in order to be able to provide correct timestamps to the TalkToProLab.sendStimulusEvent() and TalkToProLab.sendCustomEvent() functions. We have not implemented this ourselves as we consider it to be a niche use case. The authors can provide guidance to anyone looking to implement such synchronization for using TalkToProLab in multi-machine setups.

It should be noted that TalkToProLab can be used in parallel with Titta’s data stream recording interface and data saving functionality. It is thus possible to record and store data to file using both Titta and Pro Lab, for instance if the researcher wants to use Tobii Pro Lab to view replays of the recordings, but wants access to the gaze position and other data from the files saved by Titta’s saveData() for further analysis, so that Pro Lab’s manual export functionality does not have to be used. It is also possible to use gaze data in real-time while recording it with Pro Lab.

### Dummy mode

To enable development of experiments using Titta and TalkToProLab without access to the eye tracker or the computer with the Tobii Pro Lab license, Titta and TalkToProLab implement a dummy mode. Dummy mode is activated by constructing an object of the TittaDummyMode and TalkToProLabDummyMode types, or, for Titta only, by calling Titta.setDummyMode(). In dummy mode, all the same functionality as for the regular class is available except that most functions do not do anything. When in dummy mode, these functions return empty values ([]) in MATLAB and None in Python. An exception to this are the functions for reading from data streams in the Titta.buffer interface when data for the gaze stream is requested. In this case, a faked gaze sample reporting the current position of the mouse cursor is returned, to aid in implementation of gaze contingent experiments.

### MATLAB and Psychophysics Toolbox specifics

Titta has been tested with 32-bit and 64-bit MATLAB R2015b, and 64-bit MATLAB R2018b on Windows 7 and 64-bit R2019a on Windows 10 and Windows Server 2019. For the 64-bit version of MATLAB, a very recent PsychToolbox release is required, specifically at minimum the SP2 version of the “Burnout” series (the 3.0.16 release series), released on September 27th, 2019 or newer. Furthermore, for the 64-bit version, the GStreamer dependency should be correctly installed for text in the setup interface to be rendered correctly (if PsychToolbox has already been installed, execute help GStreamer in the MATLAB command line for instructions). For the 32-bit version of MATLAB, Psychtoolbox 3.0.11 must be used as it was the last version to support 32-bit MATLAB. Since this version was released in 2014, it is highly recommended to use a 64-bit installation of MATLAB to benefit from continued development of the 64-bit version of PsychToolbox.

For Linux, Titta has been tested with MATLAB R2019a on Ubuntu 18.04.3. Note that not all Tobii eye trackers are supported on Linux due to lack of drivers (e.g., at the time of writing, Tobii Pro Nano). At the time of writing, Titta does not support GNU Octave, due to several blocking bugs in the current Octave 5.1.0 release. The MEX file provided as part of Titta can be built against Octave, and could thus be used by advanced users. Octave will be supported in the future, once the outstanding bugs are resolved in a new release.

### Python and PsychoPy specifics

Titta has been tested on Windows 64-bit Windows with PsychoPy standalone 3.1.2 (Python 3.6), which comes with the required tobii_research package preinstalled. Since PsychoPy includes a graphical user interface for building experiments (PsychoPy Builder), Titta can be used to add eye tracking to any Builder experiment (see ‘example’ folder on Github).

## Example use and getting started

Titta has been designed to enable adding eye-tracking functionality to existing experiments with just a few lines of code. Follow the below steps to get started with Titta. These steps will have you install Titta, run its demos and get you set up to develop your own experiments using Titta.

1. Download Titta from https://github.com/dcnieho/Titta (MATLAB) or https://github.com/marcus-nystrom/Titta (Python) using the provided instructions and make sure that it is added to the MATLAB or Python function path so that its functionality can be accessed from MATLAB or Python scripts. For the Python version, Titta can also be installed using the command pip install git+https://github.com/marcus-nystrom/Titta.git#egg=Titta.
2. Try out Titta by launching one of the demos in the demos directory included with the Titta distribution. Take one of the below scripts, and modify it to interface with the Tobii eye tracker that you wish to use. An easy way to do so is to just run the script. If Titta does not find a Tobii Pro Spectrum (for which the demos are configured by default), it will inform you which supported eye tracker(s) it does find. Edit the demo script to connect to one of these available eye trackers. The following demos are included with Titta:
  - Readme scripts. These scripts show all the functionality of Titta in a minimal experiment consisting of two trials in which a fixation point is shown, followed by an image. Several versions of the readme script exist. readme shows Titta in its default set up and functions as a base script. Three further versions of this readme experiment have been created to showcase specific aspects of Titta functionality: readmeChangeColors demos customization of the Titta color scheme, readmeTwoScreens demos use of Titta with separate participant and operator screens, and readmeProLabIntegration demos use of the TalkToProLab class to record data for a MATLAB or Python experiment with Tobii Pro Lab. Example MATLAB code for performing fixation classification on data recorded with the readme scripts using the I2MC algorithm (Hessels et al., 2017) is also provided.
  - The Break Out! game. The breakOut script presents a gaze-controlled version of this popular computer game. Real-time gaze data is used to control the paddle in this game.
  - An antisaccade task. The antiSaccade script offers an implementation of the antisaccade protocol recommended by Antoniades et al., (2013). The antiSaccadeProLabIntegration script provides a version with Tobii Pro Lab integration.
3. To add eye tracking to your existing experiment code, copy over the required function calls from the demos. Alternatively, to get started with implementing an eye-tracking experiment from scratch, the demos may provide a good scaffold to build your experiment upon.
4. Peruse the full documentation, which is found in the readme.md file in the Titta distribution.

## Conclusion

In this article, we presented Titta, a toolbox which in combination with the PsychToolbox or PsychoPy toolboxes provides a simple and powerful way of conducting eye-tracking experiments using Tobii eye trackers. We furthermore presented the TalkToProLab tool that comes included with Titta and enables experiments implemented in MATLAB or Python to be recorded, replayed and analyzed in the Tobii Pro Lab software.

## Electronic supplementary material

Below is the link to the electronic supplementary material.

(MP4 86.6 MB)

## Footnotes

1 “Titta” is Swedish for “look!”, and was inspired by the Ikea-style of product naming.

2 See http://developer.tobiipro.com/commonconcepts/coordinatesystems.htmlhttp://developer.tobiipro.com/commonconcepts/coordinatesystems.html.

3 For PsychToolbox, on Windows the Tobii SDK and PsychToolbox timestamps correspond and thus require no further processing. On Linux, PsychToolbox uses a different system clock than the Tobii Pro SDK. Titta therefore transparently performs further remapping of PsychToolbox timestamps to the clock used by the Tobii Pro SDK.
